# Central adiposity is associated with the severity and multiligamentous involvement of ossification of the posterior longitudinal ligament

**DOI:** 10.1093/jbmrpl/ziag165

**Published:** 2026-10-13

**Authors:** Huohuo Xue, Tsutomu Endo, Yoshinao Koike, Ryo Fujita, Akinobu Nakamura, Kyu Yong Cho, Yuki Oe, M Alaa Terkawi, Masahiro Kanayama, Katsuhisa Yamada, Ken Kadoya, Hideki Sudo, Misaki Ishii, Norimasa Iwasaki

**Affiliations:** Department of Orthopedic Surgery, Hokkaido University Graduate School of Medicine, Sapporo 060-8638, Japan; Department of Orthopedic Surgery, Hokkaido University Graduate School of Medicine, Sapporo 060-8638, Japan; Spine Center, Hakodate Central General Hospital, Hakodate 040-8585, Japan; Department of Orthopedic Surgery, Hokkaido University Graduate School of Medicine, Sapporo 060-8638, Japan; Department of Orthopedic Surgery, Hokkaido University Graduate School of Medicine, Sapporo 060-8638, Japan; Department of Rheumatology, Endocrinology and Nephrology, Faculty of Medicine and Graduate School of Medicine, Hokkaido University, Sapporo 060-8638, Japan; Department of Rheumatology, Endocrinology and Nephrology, Faculty of Medicine and Graduate School of Medicine, Hokkaido University, Sapporo 060-8638, Japan; Institute of Health Science Innovation for Medical Care, Hokkaido University Hospital, Sapporo 060-8648, Japan; Department of Rheumatology, Endocrinology and Nephrology, Faculty of Medicine and Graduate School of Medicine, Hokkaido University, Sapporo 060-8638, Japan; Division of Diabetes and Endocrinology, Department of Medicine, NTT Sapporo Medical Center, Sapporo 060-0062, Japan; Department of Orthopedic Surgery, Hokkaido University Graduate School of Medicine, Sapporo 060-8638, Japan; Spine Center, Hakodate Central General Hospital, Hakodate 040-8585, Japan; Department of Orthopedic Surgery, Hokkaido University Graduate School of Medicine, Sapporo 060-8638, Japan; Department of Orthopedic Surgery, Hokkaido University Graduate School of Medicine, Sapporo 060-8638, Japan; Department of Orthopedic Surgery, Hokkaido University Graduate School of Medicine, Sapporo 060-8638, Japan; Spine Center, Hakodate Central General Hospital, Hakodate 040-8585, Japan; Department of Orthopedic Surgery, Hokkaido University Graduate School of Medicine, Sapporo 060-8638, Japan

**Keywords:** OPLL, central adiposity, visceral fat, multiligamentous ossification, metabolic phenotype, body roundness index, obesity, obesity-related inflammation

## Abstract

Ossification of the posterior longitudinal ligament (OPLL) is increasingly recognized as a metabolic ossification phenotype characterized by obesity-related abnormalities, particularly visceral adiposity. Although visceral fat has been implicated in the development of OPLL, its relationship with ossification severity and multiligamentous involvement remains unclear. This study aimed to examine whether anthropometric indices reflecting central adiposity show consistent associations with the burden of spinal ligament ossification. We retrospectively analyzed 252 individuals, including 163 patients with OPLL and 89 controls. Anthropometric indices—BMI and body roundness index (BRI)—were evaluated alongside CT-based measurements of visceral and subcutaneous fat areas. Ossification severity was quantified using a validated CT-based ossification index encompassing OPLL, ossification of the ligamentum flavum (OLF), ossification of the anterior longitudinal ligament (OALL), and ossification of the supra/interspinous ligaments (OSIL). Logistic regression, spline modeling, and sex-stratified analyses were performed. Patients with OPLL, particularly those with diffuse-type disease, exhibited significantly greater visceral adiposity and higher BMI and BRI values than controls. Both BMI and BRI were associated with the presence of diffuse OPLL, whereas their associations with localized OPLL were weaker. Nonlinear analyses showed that BMI was associated with ossification severity mainly within the lower BMI range, whereas BRI demonstrated generally linear associations across multiple ligamentous sites. These patterns were more evident in females. These findings suggest that anthropometric indices reflecting central adiposity may provide complementary information when evaluating the extent of spinal ligament ossification. While CT-derived visceral fat remains the direct measure of abdominal adiposity, indices, such as BRI, may offer additional context for understanding metabolic features associated with ossification burden in OPLL.

## Introduction

Ossification of the posterior longitudinal ligament (OPLL) is traditionally regarded as a localized spinal ligament disorder; however, accumulating evidence indicates that its development is strongly influenced by systemic metabolic dysfunction.^1^^,^^2^ Although OPLL most commonly affects the cervical spine in East Asian populations,^3^ it exhibits marked heterogeneity. Notably, thoracic (diffuse-type) OPLL is characterized by extensive ossification throughout the spine, often involving multiligamentous ossification including ossification of the anterior longitudinal ligament (OALL), ossification of the ligamentum flavum (OLF), and ossification of the supra/interspinous ligaments (OSIL), and is frequently accompanied by severe obesity.^3–5^ Ossification of the posterior longitudinal ligament is a major cause of myelopathy and often requires complex spinal surgery, yet surgical treatment remains largely palliative. These clinical challenges highlight the importance of understanding systemic factors that contribute to disease progression and severity.

Diffuse-type OPLL represents a distinct metabolic phenotype strongly associated with obesity-related abnormalities, such as visceral adiposity, dyslipidemia, and insulin resistance.^1^^,^^2^^,^^5–12^ A genome-wide association study using Mendelian randomization in a Japanese cohort further demonstrated that elevated BMI contributes to the development of diffuse-type OPLL.^13^ Moreover, individuals of East Asian descent are more prone to visceral fat accumulation, which predisposes them to metabolic dysfunction and low-grade inflammation.^14–19^ This ethnic predisposition suggests that visceral adiposity may be a more relevant determinant of OPLL risk than general obesity.^8^^,^^9^ Collectively, these observations support the concept that OPLL may represent a musculoskeletal manifestation of metabolic disease rather than a purely localized spinal condition. However, despite the recognized role of visceral adiposity in OPLL, whether it drives the progression or multiligamentous expansion of spinal ligament ossification remains unknown.

From the perspective of musculoskeletal loading, BMI remains a practical and widely used indicator of excess body weight and mechanical burden on the spine; however, it does not adequately capture qualitative differences in fat distribution or metabolic risk.^20–22^ Individuals with identical BMI values may have markedly different proportions of visceral and subcutaneous fat, limiting BMI’s ability to reflect metabolic risk. Given that OPLL is thought to involve dual pathogenic mechanisms—mechanical loading due to excess body weight and metabolic inflammation driven by visceral adiposity—reliance on BMI alone may underestimate or misclassify true metabolic risk.^8^^,^^9^ The body roundness index (BRI), derived from waist circumference (WC) and height, has emerged as a simple and sensitive marker of central adiposity and metabolic dysfunction.^23–25^ Body roundness index has demonstrated superior performance over BMI in identifying visceral fat accumulation and assessing metabolic abnormalities.^24–27^

Based on these considerations, we hypothesized that BRI may show a more consistent association with the overall burden of spinal ligament ossification than BMI. To evaluate this possibility, we compared BMI and BRI as alternative anthropometric indicators in relation to identical CT-based ossification indices across the entire spine. This approach allowed us to assess whether BRI provides additional or more sensitive information beyond BMI in capturing the extent of ossification severity in OPLL.

## Materials and methods

### Study design

This study was conducted as a retrospective cross-sectional analysis. Patients with OPLL were enrolled from were recruited between 2020 and 2024. The study protocol adhered to the principles of the Declaration of Helsinki (1964) and was approved by the institutional ethics committee (approval number: 2020-14). Because anonymized retrospective data were used for the analysis, the requirement for written informed consent was waived.

### Patients with OPLL

All patients with OPLL were regularly evaluated at the spine outpatient clinic of the only specialized spine care center serving a city of approximately 240 000 residents in northern Japan. Diagnosis was based on clinical symptoms of myelopathy or radiculopathy together with imaging examinations, including radiography, MRI, and CT. All patients were followed by certified spine surgeons. Consistent with previous studies, CT imaging played a critical role in assessing spinal ossification. With informed consent, patients underwent whole-spine CT to evaluate the extent and severity of ossification. Ultimately, 163 patients with OPLL (80 men and 83 women) were included.

### Control patients

Control participants were drawn from a hospital-based health examination program at the same institution where OPLL patients received clinical care. In the Japanese healthcare system, such hospital-based check-ups are routinely offered to a broad population—including employees, healthcare workers, and community residents—independent of regular outpatient clinic visits. Thus, the control group represents health-check examinees rather than clinic-based patients.

Between April 2020 and May 2021, 12 740 individuals underwent annual or biennial health examinations at this institution. Participation in trunk CT scanning was optional and selected by a subset of examinees, primarily for early cancer screening or for abdominal visceral fat assessment, and only a minority of examinees elected to undergo CT imaging. All CT scans for control participants were obtained at the same institution using the same scanner model and standardized imaging protocols as those used for the OPLL group.

Among the 12 740 examinees, 525 individuals had available axial CT images covering the cervical to pelvic regions. Of these, 249 had additionally undergone abdominal CT for visceral fat quantification. All 249 CT datasets were reviewed to assess spinal ligament ossification (OPLL, OALL, and OLF) from the cervical through LS using previously described methods.^5^^,^^8^^,^^11^ Individuals with any detectable spinal ligament ossification were excluded. The remaining 89 individuals (44 men and 45 women) without ossification were included as the control group for all analyses.

### Assessment of spinal ligament ossification

Spinal ligament ossification was evaluated using CT imaging, following previously established criteria.^3^^,^^6^^,^^7^^,^^9^^,^^10^ Ossification of the posterior longitudinal ligament was defined as ossification with a thickness of ≥2 mm. Ossification of the ligamentum flavum was identified when the thickness was ≥3 mm on axial images, when ossifications <3 mm were clearly visible, or when a mushroom-shaped ossification was present at the center of the laminae.^10^^,^^28^ Ossification of the anterior longitudinal ligament was defined as ossification with a thickness of ≥4 mm on axial images.^3^^,^^5^^,^^10^ All CT scans were independently reviewed by two board-certified spine surgeons (TE and RF), and any discrepancies were resolved by consensus. Prior to the main evaluation, both readers assessed CT images from 20 patients with OPLL, OLF, and OALL, and demonstrated high inter-rater reliability (intraclass correlation coefficient, 0.71-0.81).

### Classification of OPLL and definitions of localized and diffuse types

Sagittal and axial whole-spine CT images were reviewed to determine the anatomical distribution of OPLL, following previously described methods.^6–11^ In this study, we adopted descriptive terminology that has been used in the previous studies and in Sato et al.,^9^ categorizing OPLL into two patterns based on the predominant region of involvement: (1) localized type, defined as ossification confined entirely to the cervical spine, and (2) diffuse type, defined as thoracic and/or lumbar OPLL with or without cervical extension. This localized/diffuse classification was determined solely by the anatomical distribution of OPLL and served as the primary grouping variable for between-group comparisons.

### Severity of spinal ligament ossification

The severity of ossification was quantified using indices based on the method originally proposed by Kawaguchi et al.^29^ and modified in later studies.^6^^,^^7^ One point was assigned for the presence of ossification at each vertebral or intervertebral level for OPLL, OALL, OLF, and OSIL. Lesions ≥5 mm in thickness were assigned double points to account for greater ossification magnitude. The total ossification (OS) index was defined as the cumulative score across all four ligaments. Representative scoring examples are shown in Figure S1A and B.

### Relationship between disease classification and ligament-specific ossification

To clarify the relationship between disease classification and ligament-specific ossification, the localized/diffuse grouping was determined solely by the anatomical distribution of OPLL, whereas OALL, OLF, and OSIL were evaluated independently using the ossification index scoring system. Thus, the classification captures the regional pattern of OPLL involvement, while the ossification indices quantify the extent of ossification across all four ligaments.

Diffuse-type OPLL was frequently accompanied by multiligamentous ossification, and higher OS indices in this group were attributable to combined contributions from OPLL, OALL, OLF, and OSIL, rather than dominance of a single ligament.^5–11^ Prior evidence also shows that diffuse OPLL is frequently associated with multiregional ligament ossification and metabolic comorbidities, such as type 2 diabetes (T2D), dyslipidemia, and severe obesity.^1^^,^^5–12^ Representative examples of the scoring procedure and multiligament involvement are shown in Figure S1.

### Measurement of fat area using CT

Waist circumference (cm), total fat area (TFA) (cm^2^), visceral fat area (VFA) (cm^2^), subcutaneous fat area (SFA) (cm^2^), and the visceral-to-subcutaneous fat area ratio (V/S ratio) were quantified using non-contrast axial CT images obtained at the level of the umbilicus, following previously described methods. Semi-automatic measurements were performed by experienced CT technicians. Visceral and subcutaneous fat areas were quantified using identical CT-based measurement procedures in both OPLL and control participants. Representative CT images illustrating visceral and subcutaneous fat distribution are provided in Figure 1C and D.

**Figure 1:**
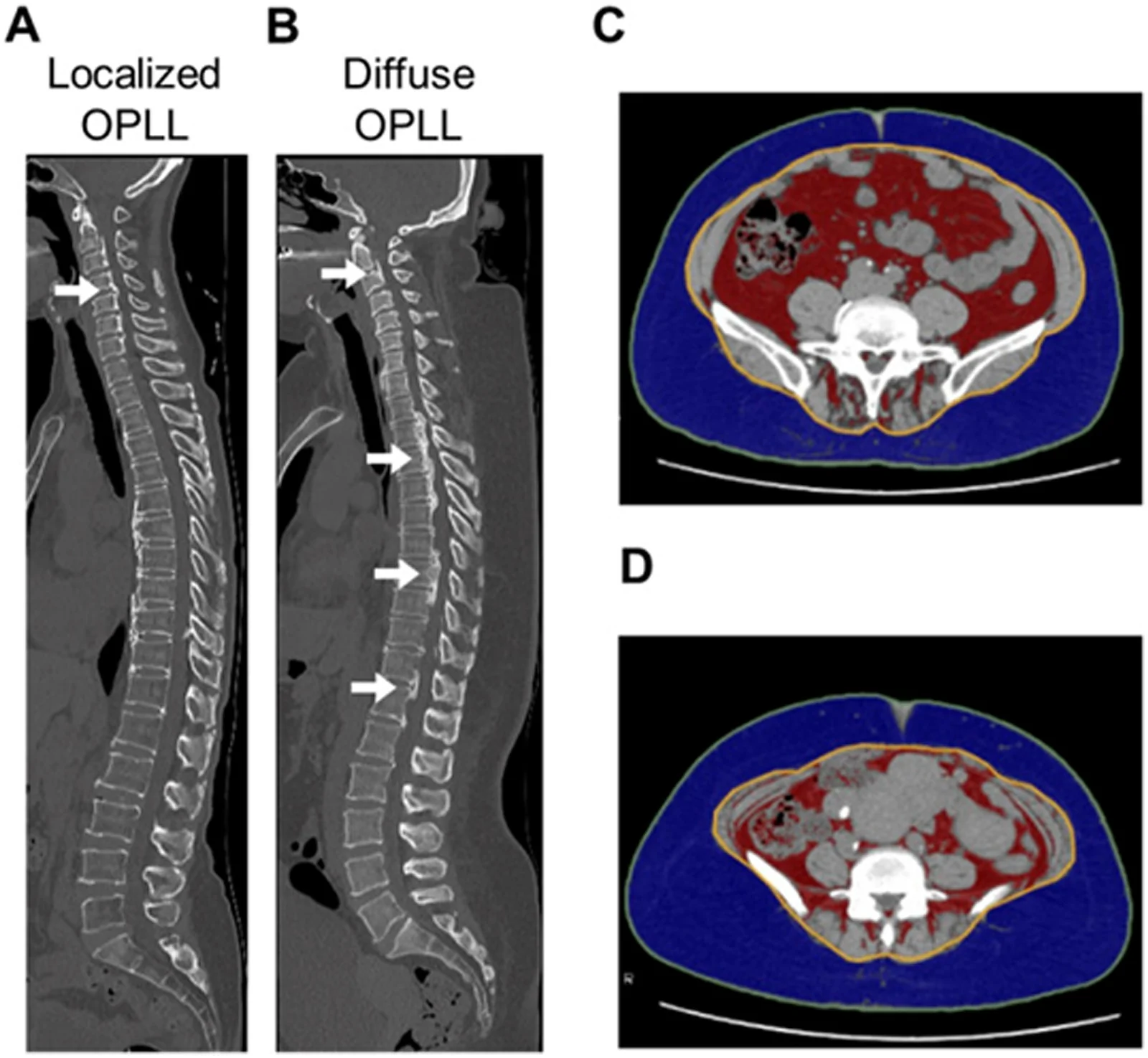
Representative cases of OPLL and visceral fat obesity shown on CT images. (A) Localized OPLL: OPLL present only in the cervical spine (arrow). (B) Diffuse OPLL: OPLL present throughout the spine, including the cervical, thoracic, and thoracolumbar spine (arrows). (C) Representative example of an abdominal CT image of a patient with obesity and visceral fat. BMI, 33.7 kg/m^2^; total fat area, 476 cm^2^; visceral fat area, 156 cm^2^; subcutaneous fat area, 319 cm^2^; V/S ratio, 0.49. (D) Representative example of an abdominal CT image of a patient with obesity but without visceral fat. BMI, 29.0 kg/m^2^; total fat area, 399 cm^2^; visceral fat area, 59 cm^2^; subcutaneous fat area, 340 cm^2^; V/S ratio, 0.17. OPLL, ossification of the posterior longitudinal ligament; V/S ratio, visceral/subcutaneous fat area ratio. (Figure reproduced from Sato et al., the journal of Bone & Joint Surgery Open Access, 2025, licensed under CC BY 4.0.)

### Demographic characteristics, comorbidities, and laboratory data

Demographic characteristics—including age, sex, height (cm), weight (kg), comorbidities (such as hypertension, T2D, and dyslipidemia, and renal disease), and current medications—were obtained using structured questionnaires. Fasting blood samples were collected for biochemical measurements, including aspartate aminotransferase (AST), alanine aminotransferase (ALT), platelet count, fasting blood glucose, glycated hemoglobin A1c (HbA1c), triglycerides (TG), high-density lipoprotein cholesterol (HDL-C), low-density lipoprotein cholesterol (LDL-C), and LDL-C/HDL-C ratio.

### The formulas for calculating BRI and BMI

Body roundness index was calculated according to the formula proposed by Thomas et al.^30^


\begin{align*} \mathbf{BRI}=\,&\mathbf{364.2}-\mathbf{365.5}\cdotp \\ &\sqrt{\mathbf{1}-{\left(\frac{\mathbf{waist}\kern0.17em \mathbf{circumference}\;\left(\mathbf{m}\right)}{\mathbf{2}\boldsymbol{\pi }}\right)}^{\mathbf{2}}\cdotp \frac{\mathbf{1}}{{\left[\mathbf{height}\;\left(\mathbf{m}\right)\right]}^{\mathbf{2}}}}. \end{align*}

BMI was calculated according to the following formula:


$$ \mathbf{BMI}=\frac{\mathbf{Weight}\ \left(\mathbf{kg}\right)}{{\left[\mathbf{height}\;\left(\mathbf{m}\right)\right]}^{\mathbf{2}}}. $$

### Multivariable logistic regression analysis

To identify and compare the associations of BMI and BRI with the risk of the development of OPLL (localized and diffuse types), two separate multivariable logistic regression models were constructed. One model included age, sex, BMI, dyslipidemia, T2D, VFA, and V/S ratio, while the other included age, sex, BRI, dyslipidemia, T2D, VFA, and V/S ratio. Standardized odds ratios (ORs) were calculated to enable comparison of effect sizes between the BMI-based and BRI-based models.

### Linear regression and restricted cubic spline modeling

To evaluate the effects of BMI and BRI on the progression of spinal ligament ossification, linear regression models were first constructed for the ossification indices of different spinal ligament sites. Standardized β coefficients were used to compare the effect sizes of independent variables across models, while model performance was assessed using the likelihood ratio tests to determine whether the linearity assumption was satisfied.

For models in which linearity was violated, restricted cubic spline (RCS) models were applied. Prior to model fitting, we performed sensitivity analyses to examine the robustness of potential nonlinear associations. Restricted cubic spline models with 3, 4, 5, and 6 knots were constructed, and model fit was compared using Akaike’s information criterion (AIC) values. Based on the overall model performance, the RCS model with three knots was selected as the optimal specification, with knots placed at the 10th, 50th, and 90th percentiles of the variable distribution; the first and third knots were treated as linear tails. The selection of independent variables and covariates was based on prior literature and the objectives of this study, including age, sex, BMI, T2D, dyslipidemia, VFA, and the V/S ratio, with the addition of BRI as a key variable of interest in the present analysis.^5^^,^^6^^,^^8–12^ The dependent variables were the respective ossification indices. Standardized β values across different BMI ranges were then calculated to quantify the relative contribution of each variable to the ossification burden.

### Comparative analysis of anthropometric indices

To evaluate whether the added computational complexity of BRI provides any practical advantage over simpler anthropometric indices, we conducted a comparative analysis using the same covariate-adjusted linear/RCS regression framework applied in the main models. For each ossification index and sex stratum, three parallel models were constructed using BMI, BRI, or WC as the independent variable of interest. All models included identical covariates: age, dyslipidemia, T2D, VFA, and the V/S ratio.

Linearity was formally assessed for each predictor. BMI violated the linearity assumption in several models and was therefore modeled using RCS. In contrast, both BRI and WC satisfied the linearity assumption across all models and were modeled as linear terms. All three specifications were treated within the same regression framework, allowing direct comparison of *p*-values and AIC values across models. To compare the relative associations of BMI, BRI, and WC with ossification severity, *p*-values for each predictor were examined across the 3 models (Figure S2A). Model performance was then compared using AIC values (Figure S2B).

### Sample size estimation

Because no comparable studies were available, sample size was estimated from the expected difference in OPLL prevalence between individuals with and without visceral fat. The prevalence of OPLL in Japanese adults is approximately 5%.^3^ Based on prior reports on OPLL occurrence across visceral fat strata,^8^ we assumed OPLL detection rates of 25% in the visceral fat group and 10% in the non-visceral fat group (α = .05, power = 0.80, 1:1 allocation), yielding a required sample size of 200. To ensure adequate power for multivariable analysis, we applied the events-per-variable (EPV) criterion (7 variables × EPV 10 = 70 events). The final dataset included 252 participants with 163 OPLL events, exceeding the EPV threshold and supporting the model.

### Handling of missing data

Missing data were observed for the following variables: WC 4.9% (8/163), platelet count 3.7% (6/163), HbA1c 39.3% (64/163), AST 3.7% (6/163), ALT 3.7% (6/163), TG 5.5% (9/163), HDL-C 5.5% (9/163), LDL-C 5.5% (9/163), dyslipidemia 3.1% (5/163), and kidney disease 0.6% (1/163). As key variables, such as dyslipidemia, had <5% missingness, complete-case analysis was applied for critical variables. A total of 163 cases were included in the final analysis.

### Statistical analysis

All statistical analyses were performed using SPSS version 23.0 (IBM). The Shapiro–Wilk test was used to assess normality. Continuous variables were expressed as mean ± SD or median (range). Between-group comparisons were conducted using independent-samples *t*-tests or one-way analysis of variance (one-way ANOVA) for normally distributed variables, and Mann–Whitney U tests for non-normally distributed variables. Categorical variables were compared using Pearson’s chi-square test or Fisher’s exact test when appropriate.

Correlation analyses were performed using Pearson or Spearman correlation coefficients, depending on the distribution of the variables. Logistic regression analyses were used to assess risk relationships between binary outcomes, and effect sizes were compared using standardized ORs. Linear regression analyses were performed to evaluate associations between continuous variables, and regression coefficients (β) with 95% CIs were reported. Multicollinearity was assessed using variance inflation factors (VIF), with a threshold of 10 indicating collinearity; variables exceeding this threshold were excluded from the models. Restricted cubic spline modeling and curve visualization were performed using R software (version 4.2.2; R Foundation for Statistical Computing) and relevant packages including rms, dplyr, and ggplot2. Statistical significance was defined as *p* < .05 for 2-group comparisons and *p* < .016 for 3-group comparisons after Bonferroni correction (0.05/3).

## Results

### Metabolic characteristics of localized and diffuse OPLL

Baseline characteristics are shown in Table 1. Patients with OPLL had higher BMI values compared with controls (control: 23.1, localized OPLL: 25.6, and diffuse OPLL: 27.5 kg/m^2^; *p* < .016). Similarly, BRI progressively increased across groups (control: 3.4, localized OPLL: 4.4, and diffuse OPLL: 5.5; *p* < .016). Visceral fat area was significantly greater in both localized and diffuse OPLL groups compared with controls (*p* < .016). In addition, diffuse OPLL had the largest SFA, as shown in Table 1 (*p* < .016).

**Table 1:** Comparison of baseline data.

Variable	Control (*n* = 89)	Localized OPLL (*n* = 49)	Diffuse OPLL (*n* = 114)
**Age**	50 (38-77)	70 (48-85)^*^	68 (31-86)^*^
**Male (%)**	49.4 (44)	79.5 (39)^*^	35.9 (41)^‡^
**BMI (kg/m** ^**2**^ **)**	23.1 (18.6-32.0)	25.6 (18.8-42.6)^*^	27.5 (16.0-50.9)^*^^,^^‡^
**BRI**	3.4 ± 1.1	4.4 ± 1.3^*^	5.5 ± 1.9^*^^,^^‡^
**Comorbidities (%)**			
**Hypertension**	14.6 (13)	79.5 (39)^*^	58.7 (67)^*^^,^^‡^
**T2D**	3.3 (3)	36.7 (18)^*^	39.4 (45)^*^
**Dyslipidemia**	52.8 (47)	79.5 (39)^*^	75.4 (86)^*^
**Renal disease**	0 (0)	6.1 (3)^*^	3.5 (4)
**Serum data**			
**Platelet (10**^**9**^**/L)**	24.0 (14.8-45.1)	23.1 (11.0-36.9)	21.2 (5.3-47.1)^*^
**AST (U/L)**	20.0 (13-58)	22.0 (13-63)	22.0 (9-63)
**ALT (U/L)**	20.0 (9-68)	20.5 (10–53)	20.0 (5–90)
**TG (mg/dL)**	210.0 (140-327)	136.5 (44-358)^*^	128.5 (39-412)^*^
**HDL-C (mg/dL)**	59.5 (30-97)	49.0 (31-102)^*^	56.0 (30-96)^†^
**LDL-C (mg/dL)**	121.0 (57-249)	100.5 (45-189)^*^	109.0 (42-208)^*^
**LDL-C/HDL-C ratio**	0.5 (0.2-1.2)	0.5 (0.2-1.1)	0.5 (0.2–1.6)
**Waist circumference (cm)**	83.4 ± 8.4	90.5 ± 10.7	93.8 ± 14.3^*^
**Fat area (cm** ^**2**^ **)**			
**Total fat**	250.9 (15.3-569.5)	311.0 (44.3-685.8)	354.1 (34.6-994.8)^*^
**Visceral fat**	92.5 ± 54.8	161.4 ± 81.1^*^	166.1 ± 84.0^*^
**Subcutaneous fat**	155.8 (9.4-437.6)	134.1 (12.1-416.3)	185.8 (2.3-672.2)^*^^,^^‡^
**V/S ratio**	0.6 (0.1-1.9)	1.1 (0.3–2.7)^*^	0.9 (0.1-14.1)^*^^,^^‡^
**Ossification index**			
**OALL index**	0	9.0 (0–46)	5.0 (0-45)^†^
**OPLL index**	0	4.0 (1–14)	9.5 (1-53)^†^
**OLF index**	0	2.0 (0–15)	4.0 (0–19)^†^
**OSIL index**	0	0.0 (0–10)	0.0 (0–7)
**OS index**	0	19.0 (1-63)	24.0 (2-71)

Data are shown as means ± SDs, medians (minimum–maximum), or percentages (numbers).

^†^ *p* < .05 (two-group comparison), vs the localized OPLL group.

^‡^ *p* < .016 (three-group comparison), vs the localized OPLL group.

^*^ *p* < .016, vs the control group.

Abbreviations: ALT, alanine aminotransferase; AST, aspartate aminotransferase; HDL-C, high-density lipoprotein cholesterol; LDL-C, low-density lipoprotein cholesterol; OALL, ossification of the anterior longitudinal ligament; OLF, ossification of the ligamentum flavum; OPLL, ossification of the posterior longitudinal ligament; OSIL, ossification of the supra/interspinous ligament; OS, ossification; T2D, type 2 diabetes; TG, triglycerides; V/S ratio, visceral-to-subcutaneous fat area ratio.

Comorbidities were more frequent in OPLL patients. Hypertension was markedly more prevalent in OPLL overall, with the highest rate observed in localized OPLL (*p* < .016). Type 2 diabetes and dyslipidemia were also more common in OPLL groups compared with controls (*p* < .016), and renal disease was observed more frequently in localized OPLL than in controls (*p* < .016).

Regarding ossification indices, diffuse OPLL exhibited significantly higher OPLL and OLF indices compared with localized OPLL (OPLL: 4.0 vs 9.5, *p* < .001; OLF: 2.0 vs 4.0, *p* = .002). The OSIL index did not differ significantly between the groups (*p* = .60). Although the OS index tended to be higher in diffuse OPLL (19.0 vs 24.0), the difference was not significant (*p* = .12).

### Correlations of BMI and BRI with fat distribution

BMI and BRI were strongly correlated in both sexes (male: *r* = 0.87; female: *r* = 0.89) (Figure 2). Among males, BRI demonstrated stronger associations with fat distribution compared with BMI. Specifically, BRI showed a higher correlation with VFA (*r* = 0.71 vs 0.59) and TFA (*r* = 0.88 vs 0.82). For SFA, the correlations were comparable (*r* = 0.78 vs 0.79).

**Figure 2:**
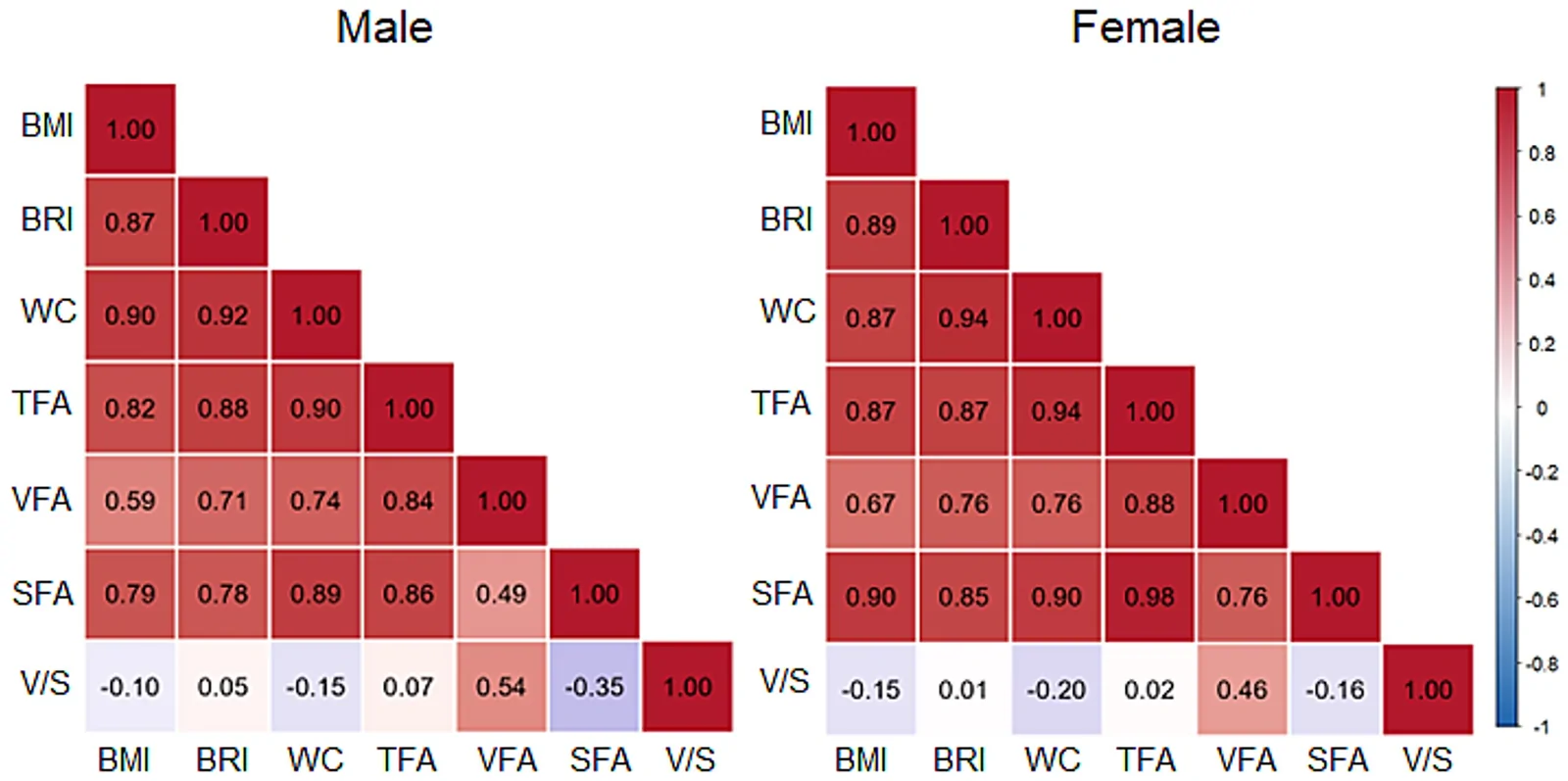
Correlation analysis of obesity-related indices in males and females. The heatmaps illustrate the correlation coefficients among BMI, BRI, WC, TFA, VFA, SFA, and the V/S ratio. In both sexes, BMI, BRI, WC, TFA, VFA, and SFA show strong positive correlations, whereas the V/S ratio demonstrates weaker associations with other adiposity parameters. Abbreviations: BRI, body roundness index; SFA, subcutaneous fat area; TFA, total fat area; VFA, visceral fat area; V/S ratio, visceral/subcutaneous fat area ratio; WC, waist circumference.

Among females, BRI also exhibited stronger correlations with VFA (*r* = 0.76 vs 0.67), while correlations with TFA were similar between BMI and BRI (*r* = 0.87 vs 0.87). For SFA, BMI showed a slightly stronger correlation than BRI (*r* = 0.90 vs 0.85). Waist circumference demonstrated correlations with VFA comparable to those of BRI in both sexes (male: *r* = 0.74; female: *r* = 0.76), indicating that WC and BRI capture central adiposity to a similar extent.

### Associations of BMI and BRI with OPLL subtype (localized vs diffuse)

Two multivariable logistic regression models were constructed to compare the effects of BMI and BRI on the development of localized and diffuse OPLL.

For localized OPLL, age and T2D were significant risk factors in both models (*p* < .05) (Figure 3). The estimated risk associated with BRI was slightly higher than that of BMI (OR: 3.9 vs 2.6), but the difference was not statistically significant.

**Figure 3:**
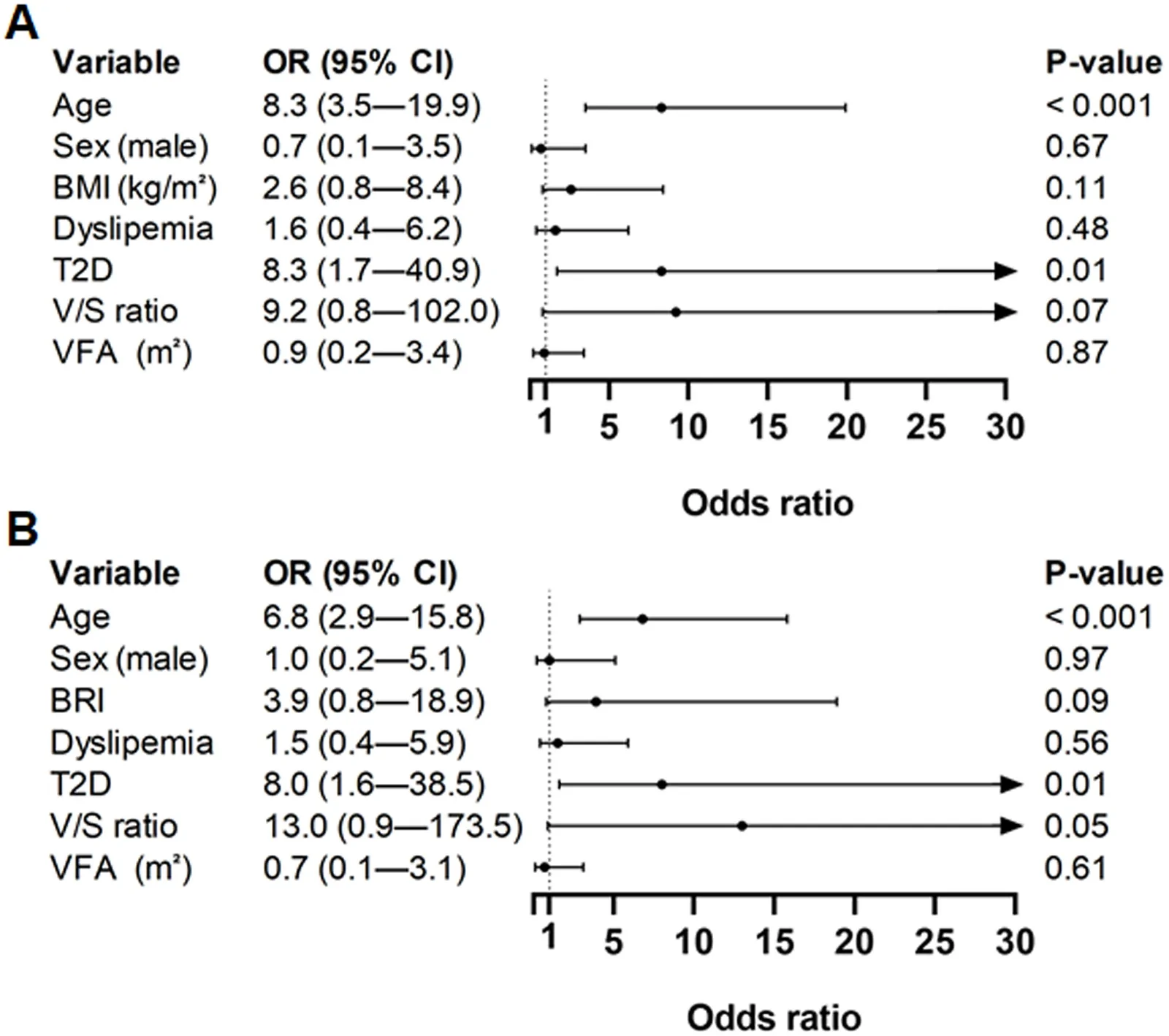
Comparison of the effect sizes of BMI and BRI on the development of localized OPLL. Bar plots represent the effect size of each variable on OPLL. Error bars represent 95% CI of the effects. (A) BMI model. (B) BRI model. OPLL, ossification of the posterior longitudinal ligament; Abbreviations: BRI, body roundness index; T2D, type 2 diabetes; VFA, visceral fat area.

For diffuse OPLL, age, T2D, and the V/S ratio were significant risk factors in both models (*p* < .05) (Figure 4). In the BMI-based model, sex emerged as a significant modifier (OR: 0.2 [0.1-0.5], *p* = .01), whereas this association did not reach significance in the BRI-based model (OR: 0.4 [0.1-1.1], *p* = .08). Both BMI and BRI were significant associated factors for the development of diffuse OPLL, with BMI showing a stronger effect than BRI (OR: 12.2 vs 9.4, *p* < .001).

**Figure 4:**
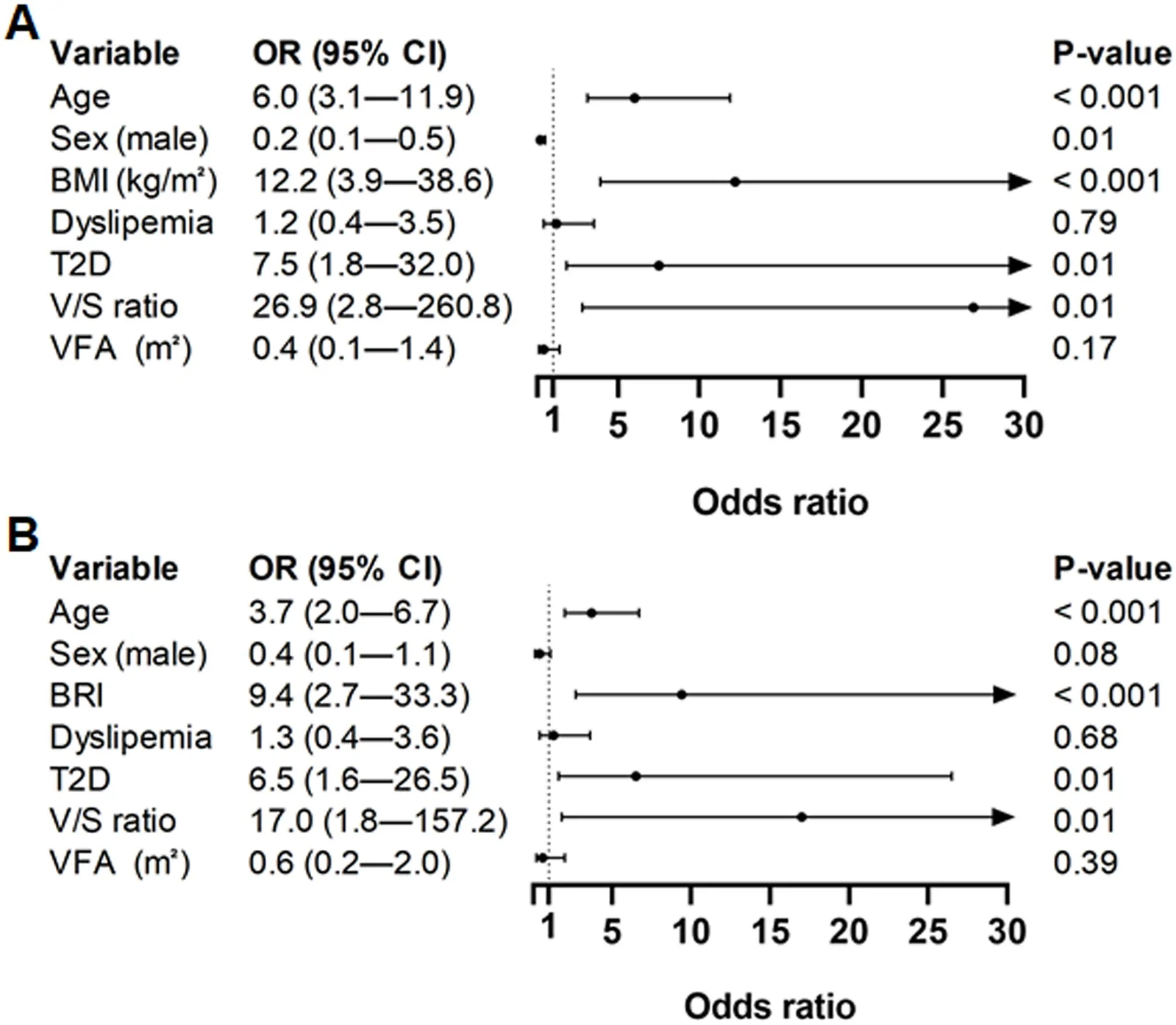
Comparison of the effect sizes of BMI and BRI on the development of diffuse OPLL. Bar plots represent the effect size of each variable on OPLL. Error bars represent 95% CI of the effects. (A) BMI model. (B) BRI model. Abbreviations: OPLL, ossification of the posterior longitudinal ligament; BRI, body roundness index; T2D, type 2 diabetes; VFA, visceral fat area.

### Comparative effects of BMI and BRI on ligament ossification severity

Sex-stratified analyses were performed to quantify ossification severity (Figures 5 and 6). For the OALL index, both BMI and BRI showed linear associations, with negative trends in males and positive trends in females; however, none reached statistical significance.

**Figure 5:**
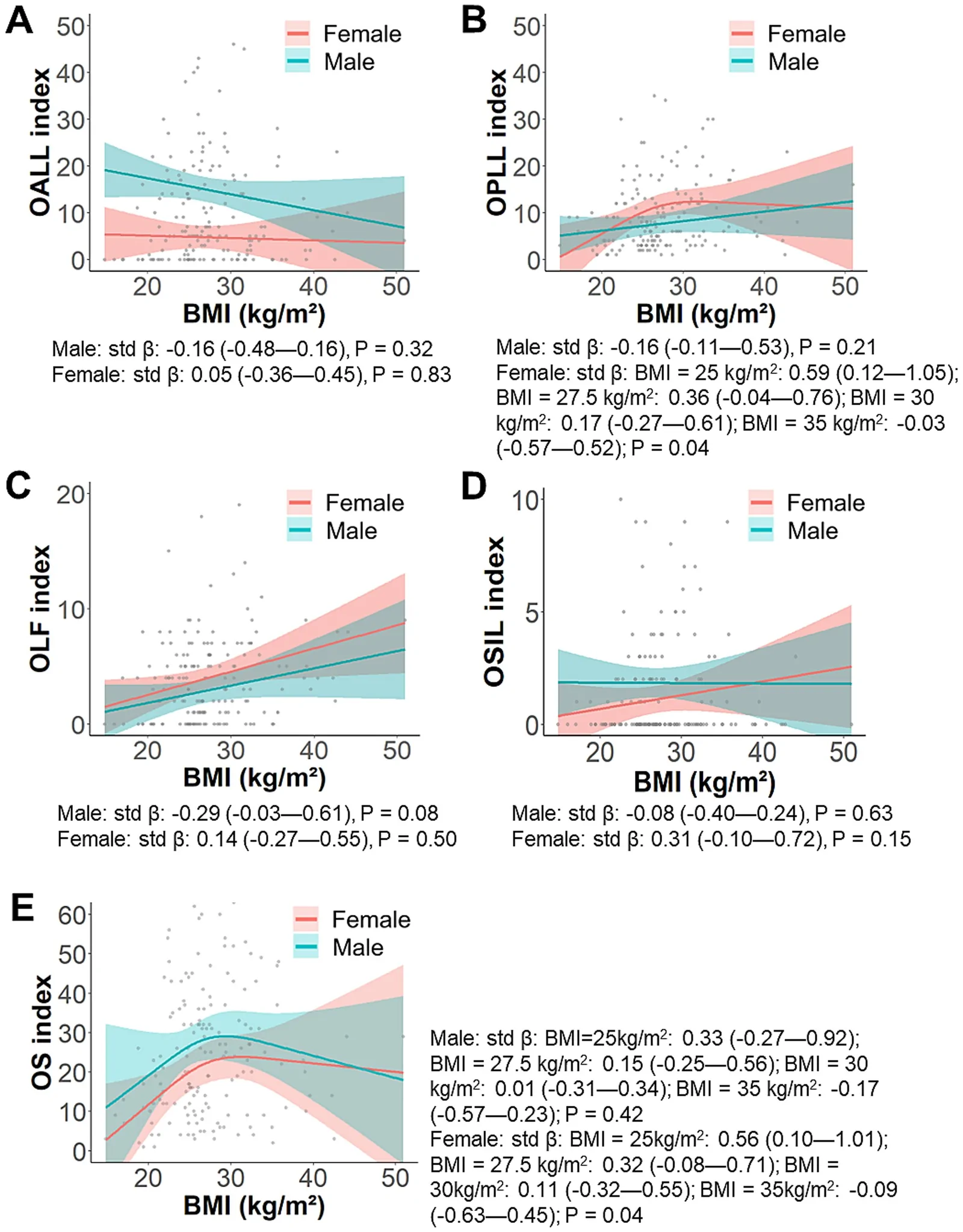
Sex-specific associations between BMI and various spinal ligament ossification indices. Trend lines illustrate the relationships between BMI and 5 types of spinal ligament ossification indices in males and females. Shaded areas indicate the 95% CIs, and standardized β coefficients and *p*-values are calculated for each interval. (A) OALL index. (B) OPLL index. (C) OLF index. (D) OSIL index. (E) OS index. Abbreviations: OALL, ossification of the anterior longitudinal ligament; OLF, ossification of the ligamentum flavum; OPLL, ossification of the posterior longitudinal ligament; OSIL, ossification of the supra/interspinous ligaments; OS, total ossification index.

**Figure 6:**
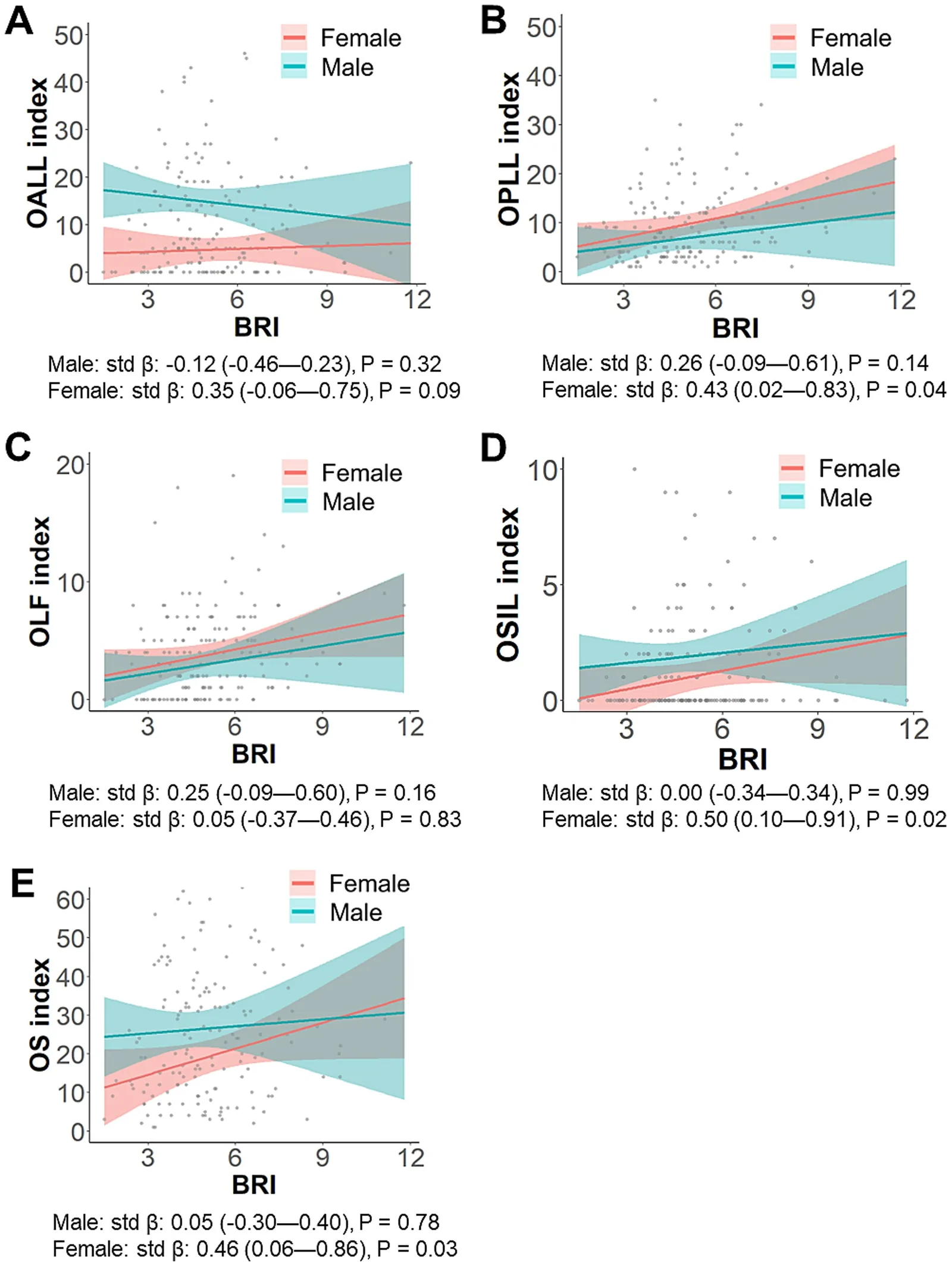
Sex-specific associations between BRI and various spinal ligament ossification indices. Trend lines illustrate the relationships between BRI and 5 types of spinal ligament ossification indices in males and females. Shaded areas indicate the 95% CIs, and standardized β coefficients and *p*-values are calculated for each interval. (A) OALL index. (B) OPLL index. (C) OLF index. (D) OSIL index. (E) OS index. Abbreviations: OALL, ossification of the anterior longitudinal ligament; OLF, ossification of the ligamentum flavum; OPLL, ossification of the posterior longitudinal ligament; OSIL, ossification of the supra/interspinous ligaments; OS, total ossification.

For the OPLL index, sex-specific differences were evident. In females, BMI exhibited a significant nonlinear association characterized by a plateau effect and declining standardized β values from .59 at BMI 25 kg/m^2^ to −.03 at BMI 35 kg/m^2^ (*p* = .04; detailed values are shown in Figure 5B). In contrast, BRI demonstrated a stable and significant linear association across the full range of OPLL severity (standardized β: .43 [0.02-0.83], *p* = .04) (Figure 6B). In males, neither BMI nor BRI showed significant associations (Figures 5B and 6B).

For the OLF index, both BMI and BRI showed positive but modest associations with ossification severity. In males, the association with BMI approached but did not reach statistical significance (standardized β: .29 [−0.03-0.61], *p* = .08), whereas BRI demonstrated a similarly weak and non-significant trend (standardized β: .25 [−0.09-0.60], *p* = .16). In females, neither BMI nor BRI showed significant associations (Figures 5C and 6C).

For the OSIL index, sex-specific differences were more pronounced. In females, BRI showed a significant association with ossification severity (standardized β: .50 [0.10-0.91], *p* = .02), whereas BMI did not (standardized β: .31 [−0.10 to 0.72], *p* = .15). In males, neither BMI nor BRI demonstrated statistically significant associations with OSIL (Figures 5D and 6D).

For the OS index, BMI demonstrated nonlinear characteristics in both sexes, with a plateau effect and a shift from positive to negative standardized β values (males: .33 at BMI 25 kg/m^2^ to −.17 at BMI 35 kg/m^2^, *p* = .42; females: .56 at BMI 25 kg/m^2^ to −.09 at BMI 35 kg/m^2^, *p* = .04; detailed values are shown in Figure 5E). In contrast, BRI maintained consistent linear associations, and a significant relationship between BRI and the OS index was observed in females (standardized β: .46 [0.06-0.86], *p* = .03) (Figure 6E).

### Additional comparative analyses of BMI, BRI, and WC

To compare the explanatory performance of BMI, BRI, and WC, we visualized the *p*-values from all RCS regression models (Figure S2A). BMI and BRI showed statistically significant associations with several ligament-specific ossification indices (*p* < .05), whereas WC demonstrated fewer significant associations. However, the pattern of significance varied across predictors and ligament sites. Given the strong correlations among BMI, BRI, and WC, these inconsistencies likely reflect multicollinearity. We next compared the AIC values for all spline-based models (Figure S2B). Akaike’s information criterion differences across BMI-, BRI-, and waist-circumference-based models were consistently within 0-3 for all ligament-specific indices, indicating no meaningful difference in overall model fit or stability. Overall, BMI, BRI, and WC exhibited comparable explanatory performance for ossification severity.

## Discussion

In this study, patients with OPLL—particularly those with diffuse-type disease—exhibited more pronounced metabolic abnormalities than controls, including elevated BMI, BRI, VFA, and SFA. Both BMI and BRI were significantly associated with the development of diffuse OPLL, whereas their associations with localized OPLL were weaker. Importantly, when examining ossification severity, BRI demonstrated more stable and linear associations than BMI across multiple ligamentous sites, particularly in females. These findings suggest that central adiposity may more accurately reflect the metabolic processes associated with the progression of ligamentous ossification.

BMI and BRI appear to capture distinct aspects of OPLL pathophysiology. BMI reflects overall body mass, integrating both fat and lean components,^22^ and its association with ossification severity at lower BMI levels may be consistent with load-driven stimulation of osteogenic pathways.^31^^,^^32^ However, prior studies have shown that the relationship between BMI and visceral adiposity weakens in the higher BMI range, where metabolic risk becomes increasingly independent of body weight.^20^^,^^33^^,^^34^ This may explain the plateau observed around 30 kg/m^2^ in our nonlinear analyses: once mechanical loading reaches a threshold, further increases in visceral adiposity are no longer captured by BMI. In contrast, BRI reflects central adiposity and more closely captures visceral fat accumulation, with several validation studies demonstrating a near-proportional relationship between BRI and visceral adipose tissue.^25^^,^^30^ Because visceral adiposity is strongly linked to obesity-related metaflammation driven by adipose-derived cytokines,^35^^,^^36^ the continuous linear association between BRI and ossification severity suggests that metabolic pathways—rather than mechanical loading alone—contribute substantially to the progression of spinal ligament ossification.

Importantly, our analyses comparing BMI, BRI, and WC showed that these anthropometric indices exhibited largely comparable explanatory performance for ligament-specific ossification. Although BMI and BRI reached statistical significance in several models, whereas WC did so less frequently, the overall pattern of significance varied across predictors and ligament sites. Given the strong correlations among BMI, BRI, and WC, these inconsistencies likely reflect multicollinearity rather than true differences in explanatory ability. Consistent with this interpretation, AIC values for all spline-based models indicated no meaningful difference in model fit or stability. Thus, BRI should not be interpreted as statistically outperforming WC or BMI. However, BRI may offer practical utility by addressing the limitations of BMI, which has limited ability to assess visceral fat levels.^20^^,^^33^^,^^34^ In contrast, BRI incorporates height-based scaling and body roundness, allowing it to represent central adiposity in a manner comparable to WC. Given that central adiposity is strongly implicated in the metabolic phenotype of OPLL, BRI may serve as a practical surrogate marker for central adiposity.

Diffuse OPLL is known to occur more frequently in women, and sex-related differences in ossification susceptibility have been described,^3^ although the underlying mechanisms remain uncertain. Based on current knowledge of adiposity-related inflammation, we speculate that post-menopausal increases in visceral fat, heightened metaflammatory responses, and reduced estrogen-mediated anti-inflammatory signaling may help explain the greater ossification burden observed in women.^37–39^ These potential mechanisms should be regarded as hypothetical, and because our nonlinear analyses demonstrated broadly similar trends across sexes, further research is needed to clarify the extent to which sex-specific metabolic factors influence ossification progression.

Mechanistically, both mechanical and metabolic factors likely interact to promote ectopic ossification in OPLL. Excessive mechanical loading may activate mechanotransduction pathways such as Piezo1, yes-associated protein (YAP) and transcriptional co-activator with PDZ-binding motif (TAZ), and mitogen-activated protein kinase (MAPK)/extracellular signal-regulated kinase (ERK),^40–42^ which stimulate osteogenic signaling in ligamentous tissues. As ossification progresses and the ligament becomes increasingly stiff, opportunities for load-driven mechanotransduction may diminish,^43^^,^^44^ shifting the relative contribution toward metabolic influences. In parallel, adipose-derived cytokines—including interleukin-6, leptin, and resistin—may amplify macrophage-mediated inflammatory pathways, such as NF-κB, transforming growth factor-β/Smad2/3, and Wnt/β-catenin.^45–50^ Activation of these pathways can enhance osteogenic activity in ligament-derived progenitor cells, promoting pro-ossific changes that may be associated with a greater ossification burden.^1^^,^^2^ Taken together, these converging mechanical and metabolic influences may provide a biologically plausible framework for understanding the association between visceral adiposity to the severity and multiligamentous expansion of ectopic spinal ossification.

We also quantified OLF, OALL, and OSIL because diffuse-type OPLL frequently coexists with multiligamentous ossification, reflecting a broader systemic ossification phenotype rather than a process limited to the posterior longitudinal ligament.^3^^,^^4^^,^^29^ Assessing these structures allowed us to capture the broader burden of ectopic spinal ossification. Although OALL showed only weak associations, both OSIL and the total OS index demonstrated meaningful relationships with central adiposity—particularly in females—suggesting that metabolic influences extend beyond OPLL itself and may be associated with the multiligamentous ossification phenotype. Recent reviews have suggested that visceral adiposity and metaflammation may be associated with ligamentous ossification, but these hypotheses have not been validated using quantitative clinical data.^1^^,^^2^ Our findings may provide clinical insight into this proposed link by demonstrating, in a CT-based cohort, that indices reflecting central adiposity show stronger and more linear associations with ossification severity than BMI, particularly in diffuse-type OPLL.

This study has several limitations. First, although central adiposity indices were associated with ossification severity, causality cannot be inferred from this cross-sectional design. Second, residual confounding related to recruitment settings cannot be fully excluded. The control group consisted of health-check examinees and therefore had a younger age distribution and may also represent individuals with relatively higher health awareness than OPLL patients; however, age and sex were included as covariates in all multivariable analyses, and the metabolic profiles of controls were within the expected range for middle-aged Japanese adults. Unmeasured confounders related to recruitment setting—such as healthcare-seeking behavior, lifestyle, and socioeconomic status—may also remain. Third, although HbA1c values had substantial missingness, the diagnostic criteria for T2D used in this study did not rely solely on HbA1c. T2D status was determined based on a documented clinical diagnosis, fasting plasma glucose ≥126 mg/dL, or current use of glucose-lowering medication; however, given the high rate of missing HbA1c values, some degree of misclassification cannot be fully excluded, and caution is warranted when interpreting these findings. Fourth, sex-stratified analyses were based on relatively small subgroups, which may have limited statistical power to detect modest associations. Non-significant findings in male-specific models should not be taken as evidence of no association, and larger sex-specific cohorts will be required to clarify potential sex-dependent differences. Finally, because the study population consisted primarily of Japanese individuals, generalizability to other ethnic groups may be limited.

### Conclusion

Both BMI and BRI were associated with diffuse-type OPLL, whereas their relationships with localized OPLL were weaker. When evaluating ossification severity, BMI showed nonlinear characteristics, while BRI demonstrated generally linear associations across multiple ligamentous sites, particularly in females. These findings suggest that anthropometric indices reflecting central adiposity may provide complementary information when assessing the burden of spinal ligament ossification. Although CT-derived visceral fat remains the direct measure of abdominal adiposity, indices, such as BRI, may offer additional context for understanding metabolic features associated with ossification burden in OPLL.

## Supplementary Material

Supplemental_Figures_ziag165

## Data Availability

Individual participant data will not be shared because of ethical and institutional restrictions on patient privacy. Deidentified aggregate data (summary statistics) and study-related documents (protocol, statistical analysis plan) will be available from the corresponding author upon reasonable request.
