# Supplementary material for: Central adiposity is associated with the severity and multiligamentous involvement of ossification of the posterior longitudinal ligament
Source: JBMR Plus. 2026 Oct 13;10(12):ziag165. doi: 10.1093/jbmrpl/ziag165 (PMC13723768; doi:10.1093/jbmrpl/ziag165)
Supplement: Supplementary file 1 [file supplemental_figures_ziag165.pdf]

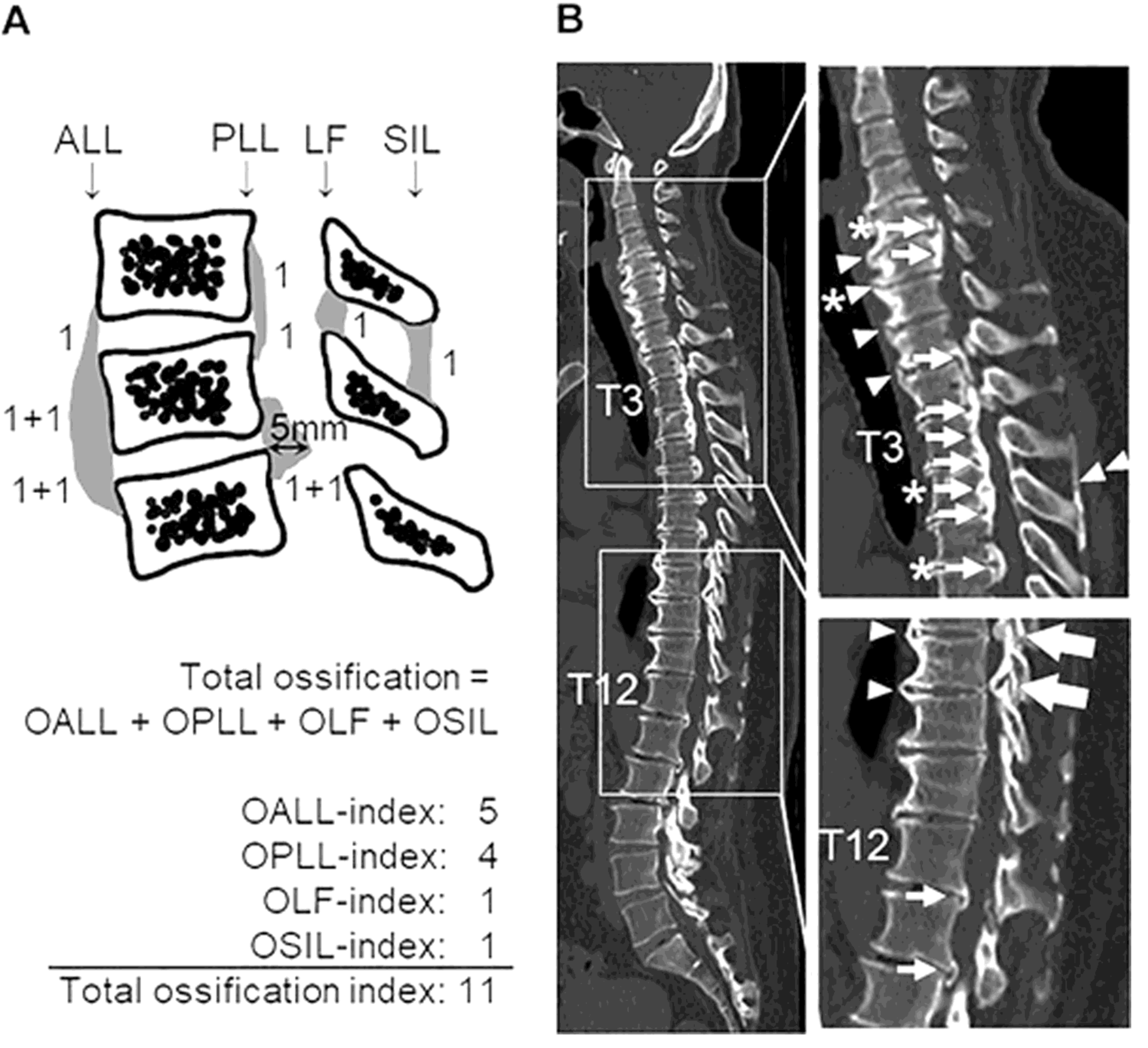

Supplement Figure 1. A representative case of ectopic ossification of spinal ligaments.

The ossification index determines the severity of ossification by scoring ligamentous ossification, including OPLL (arrow) and OLF (arrowhead) in each vertebra and intervertebral disc as 1 point. Asterisks indicate massive ossifications (thickness ≥5 mm), which are scored as 2 points.

Sagittal reconstructed CT images of the thoracic spine showing multilevel and heterogeneous ligamentous ossifications with highlighted regions of interest.

OALL, ossification of the anterior longitudinal ligament; OLF, ossification of the ligamentum flavum; OPLL, ossification of the posterior longitudinal ligament; OSIL, ossification of the supra/interspinous ligaments; ALL, the anterior longitudinal ligament; PLL, the posterior longitudinal ligament; LF, the ligamentum flavum; SIL, the supra/interspinous ligament.

Figure reproduced from Sato et al., The Journal of Bone & Joint Surgery Open Access, 2025, licensed under CC BY 4.0.

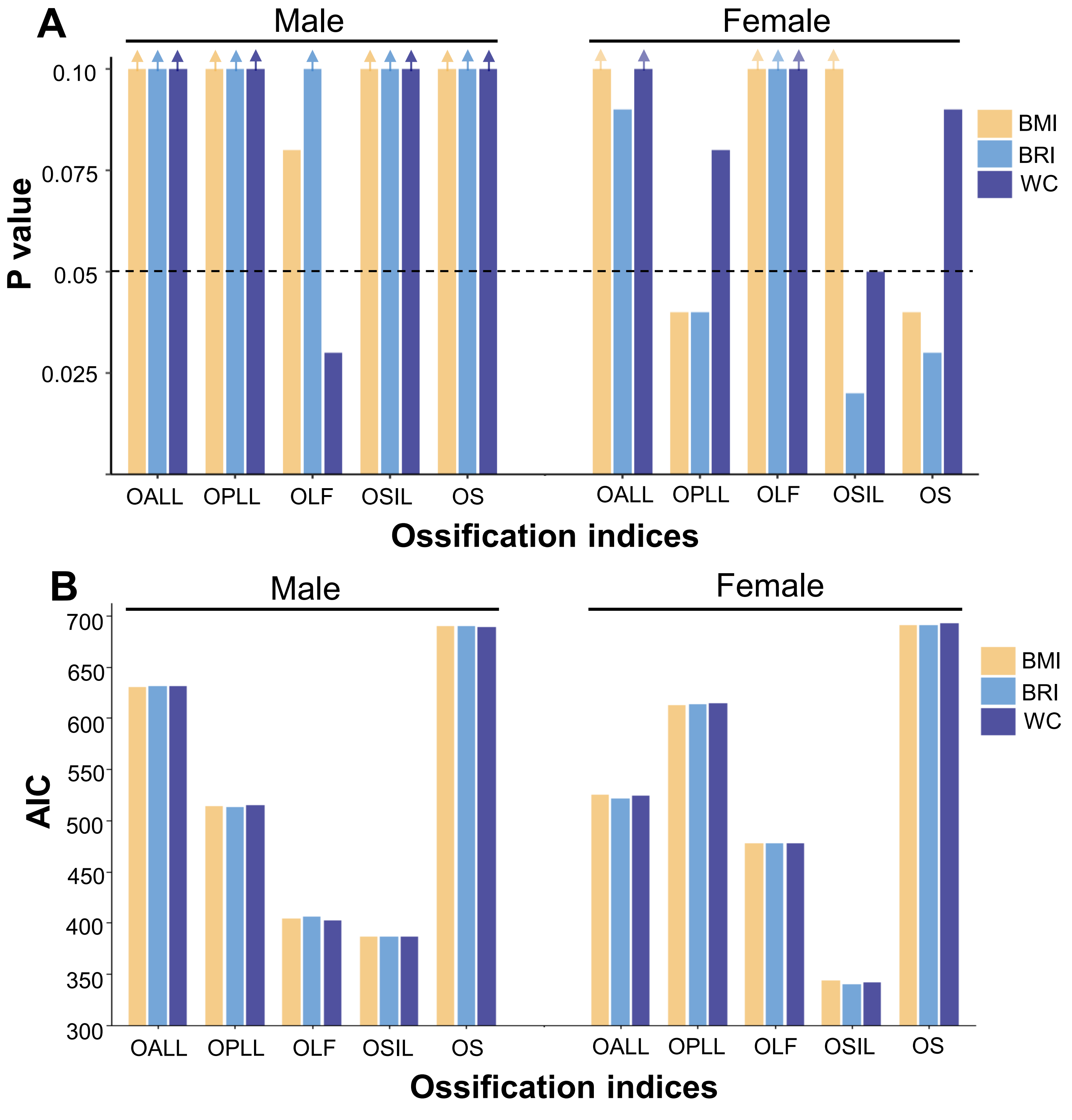

Supplement Figure 2. Comparison of P values and AIC values for BMI, BRI, and WC across ligament ossification indices by sex.

A. Bar plots show P values from covariate-adjusted linear/RCS regression models for each ossification index (OALL, OPLL, OLF, OSIL, OS), stratified by sex, comparing BMI, BRI, and WC as independent variables. The dashed horizontal line indicates P = 0.05.

B. Bar plots show AIC values from the same covariate-adjusted linear/RCS regression models for each ossification index, stratified by sex, comparing BMI, BRI, and WC as independent variables. Lower AIC values indicate better model fit; the near-identical values across the three predictors within each group indicate comparable model fit.

AIC, Akaike information criterion; BMI, body mass index; BRI, body roundness index; WC, waist circumference; RCS, restricted cubic spline; OALL, ossification of the anterior longitudinal ligament; OPLL, ossification of the posterior longitudinal ligament; OLF, ossification of the ligamentum flavum; OSIL, ossification of the supra/interspinous ligaments; OS, total ossification.
